# Inoculant of Arbuscular Mycorrhizal Fungi (*Rhizophagus clarus*) Increase Yield of Soybean and Cotton under Field Conditions

**DOI:** 10.3389/fmicb.2016.00720

**Published:** 2016-05-25

**Authors:** Martha V. T. Cely, Admilton G. de Oliveira, Vanessa F. de Freitas, Marcelo B. de Luca, André R. Barazetti, Igor M. O. dos Santos, Barbara Gionco, Guilherme V. Garcia, Cássio E. C. Prete, Galdino Andrade

**Affiliations:** ^1^Laboratório de Microbiologia, Instituto de Ciências Agrarias e Ambientais, Universidade Federal de Mato GrossoSinop, Brazil; ^2^Laboratório de Ecologia Microbiana, Departamento de Microbiologia, Universidade Estadual de LondrinaLondrina, Brazil; ^3^United Phosphorus Limited do BrazilSão Paulo, Brazil; ^4^Fazenda Escola, Departamento de Agronomia, Universidade Estadual de LondrinaLondrina, Brazil

**Keywords:** nutrient uptake, seed inoculation, rhizosphere, *Glycine max* L., *Gossypium hirsutum* L.

## Abstract

Nutrient availability is an important factor in crop production, and regular addition of chemical fertilizers is the most common practice to improve yield in agrosystems for intensive crop production. The use of some groups of microorganisms that have specific activity providing nutrients to plants is a good alternative, and arbuscular mycorrhizal fungi (AMF) enhance plant nutrition by providing especially phosphorus, improving plant growth and increasing crop production. Unfortunately, the use of AMF as an inoculant on a large scale is not yet widely used, because of several limitations in obtaining a large amount of inoculum due to several factors, such as low growth, the few species of AMF domesticated under *in vitro* conditions, and high competition with native AMF. The objective of this work was to test the infectivity of a *Rhizophagus clarus* inoculum and its effectiveness as an alternative for nutrient supply in soybean (*Glycine max* L.) and cotton (*Gossypium hirsutum* L.) when compared with conventional chemical fertilization under field conditions. The experiments were carried out in a completely randomized block design with five treatments: Fertilizer, AMF, AMF with Fertilizer, AMF with 1/2 Fertilizer, and the Control with non-inoculated and non-fertilized plants. The parameters evaluated were AMF root colonization and effect of inoculation on plant growth, nutrient absorption and yield. The results showed that AMF inoculation increased around 20 % of root colonization in both soybean and cotton; nutrients analyses in vegetal tissues showed increase of P and nitrogen content in inoculated plants, these results reflect in a higher yield. Our results showed that, AMF inoculation increase the effectiveness of fertilizer application in soybean and reduce the fertilizer dosage in cotton.

## Introduction

Nutrient availability is crucial to plant growth and crop production. This is influenced by several factors such as the chemical and physical properties of soil, climate and crop type. Crop production in tropical soils requires large amounts of chemical fertilizers, which enhance nutrient release and availability for plant nutrition (Miransari, 2011). Soybean (*Glycine max* L.) is a legume plant, of Fabaceae family. It is cultivated on large scale because has good adaptability to different soil and climatic conditions. Brazil is the second largest world producer of soybeans after the United States, and the total planted area reached 30,105 thousand hectares. Cotton (*Gossypium hirsutum* L.) is a dicotyledonous plant of Malvaceae family, also cultivated in large scale, its cultivation is an economically important activity for the country and reached around of 1,102.8 thousand hectares of planted area (IBGE, 2014). Therefore, cotton and soybean are two important crops in Brazil and, represent around 58% of the total cultivated area (CONAB, 2014) requiring a large amounts of chemicals fertilizers.

The large use of chemical fertilizers has a serious impact on the environment (Tilman et al., 2002) and the agricultural practices influence soil microorganisms greatly, decreasing soil fertility and organic matter turnover (Altieri, 1999). However, the more crucial issue for modern agriculture is that the natural reservoir of some nutrients as phosphorus (P) is decreasing in the world, leading to increase in fertilizer prices in the last decade (Cordell et al., 2009). The challenge for crop production is change to sustainable practices, by finding alternatives for increasing nutrient availability for plant nutrition as organic fertilization. Some these alternatives for organic fertilization include the use de soil microorganisms (Barrios, 2007; Miransari, 2011). Soil microorganisms play an important role by contributing significantly to nutrient availability through biochemical transformations. Some of these microorganisms act directly on plant nutrition by establishing symbiotic associations with plant roots (Bardgett, 2005). The symbiosis between arbuscular mycorrhizal fungi (AMF) and plant roots is one of the most known beneficial interactions occurring in soil (Smith and Smith, 2011), playing an important role in crop production and nutrient turnover (Andrade, 2004).

Arbuscular mycorrhizal fungi increase the uptake of soil inorganic nutrients, mainly P (Neumann and George, 2010). In addition, other benefits related to AMF are the stabilization of soil aggregates (Rillig, 2004), increased resistance to water stress (Garg and Chandel, 2010) and protection against pathogens (Jung et al., 2012). The use of biofertilizer is considered a good alternative to replace or reduce chemical fertilizer use. In example, other symbiotic microorganisms have been successfully used in soybean, and currently, *Bradyrhizobium* and other genera of symbiotic N-fixing bacteria are extensively used as biofertilizer in intensive soybean culture (Deaker et al., 2004) but not for AMF inocula.

In recent years, interest in AMF has focused on finding a viable method to optimize the production of AMF inoculum to use as inoculant in crop systems (Gianinazzi and Vosátka, 2004; Ijdo et al., 2011). The AMF inoculation in field conditions was been evaluated by some authors as Romero and Bago (2010), Pellegrino et al. (2011, 2012), and Ortas (2012) showing a high potential to increase crops yields. However, the success of AMF inoculation in agricultural soils can be determined by many factors such as species compatibility, habitat niche availability for AMF and competition with native fungi (Verbruggen et al., 2013), these aspects need to be evaluated under local conditions for a more appropriate assessment of the viability of AMF use as biofertilizer in crops.

The potential of colonization in soil of *in vitro Rhizophagus clarus* inoculum was first assessment in cotton and soybean in greenhouse conditions. No differences were found between *R. clarus in vitro* and pot culture inoculums for root colonization, plant biomass and P uptake. These results showed the successful of this AMF isolate in pure culture and the potential of this species for large-scale inoculum production (Cely, 2014).

The objective of this work was to determine the effectiveness of AMF (*R. clarus*) inoculation in two crops soybean (*Glycine max* L.) and cotton (*Gossypium hirsutum* L.), assessing its effect on plant growth, nutrient uptake and yield when compared with conventional chemical fertilization under field conditions. Our hypothesis is that AMF inoculation can be an alternative for total nutrient supply or more effective nutrient absorption, when combined with chemical fertilization.

## Materials and Methods

### Experimental Area

The experiments were carried out in Londrina city – PR, Brazil (23°55′46″ S and 51°19′11″ W) during summer (November to June). The climate is humid subtropical, with rainfall during all seasons, relative humidity around 69% and about 2,000 mm of annual precipitation, and the average summer temperature is around 29.5°C.

Two experimental areas were used (A1 and A2) with a Rhodic Ferralsol soil type according FAO (1994). Soil chemical composition and the number of indigenous AMF were determined before sowing by wet sieving and decanting (Gerdemann and Nicolson, 1963) (**Table 1**).

**Table 1:** Soil properties of the experimental areas.

Area	P (mg dm^3^)	C (g dm^3^)	pH	cmol_c_ dm^-3^							%		MP (Spores/g)
						
				Al	H^+^ Al	Ca	Mg	K	S	CEC	V	SA	
A1	12.2	18.42	4.8	0.17	6.20	5.02	1.76	0.84	7.62	13.8	55.13	2.18	3
A2	17.3	17.45	5.0	0.00	5.76	4.15	1.72	0.56	6.43	12.2	52.74	0.00	4
			
P–K: Mehlich I				Ca–Mg–Al: KCl M							pH: CaCl_2_ 0.01 M		


*S, bases sum; CEC, cation-exchange capacity; V, saturation for bases; SA, Saturation for Al; MP, mycorrhizal propagules.*

### AMF Inoculum Production and Seeds Inoculation

The *R. clarus* inoculum was produced *in vitro* conditions. The monoxenic culture was obtained using carrot (*Daucus carota* L.) Ri T-DNA transformed roots as host organs (**Supplementary Figure S1**). The *R. clarus* cultures were maintained by continuous subculture of young colonized root fragments (every 4–5 weeks at 25°C, in the dark) in modified Strullu–Romand medium (MRS; Declerck et al., 1998). Petri dishes with massive growth (mycelia and spores) of *R. clarus* and colonized roots were used as crude inoculum. The inoculation methods consist in the seeds palletization with different propagules (colonized roots, hyphae fragments, and spores) from *in vitro* pure cultures of *R. clarus* helped by an organic matrix and turf. The procedure to obtain massive inoculum and seeds inoculation is described in the patent INPI BR 10 2014 017389 7 – July 15, 2014 (Andrade et al., 2014).

### Experimental Design

#### Soybean Experiments

Two experiments were carried out with soybean, first in the harvest 2012/13 (E1), using a conventional soybean var. BRS 133 and the second in the harvest 2013/14 (E2) with a transgenic soybean var. BRS 359 RR. Both experiments were composed by the following treatments: Control (non-AMF inoculation and non-fertilizer application); Fertilizer (200 kg ha^-1^ NPK 0:20:20); AMF (*R*. *clarus* inoculation plus 65 kg ha^-1^ KCl); AMF + Fertilizer (*R. clarus* inoculation plus 200 kg ha^-1^ NPK 0:20:20); and AMF + 1/2 Fertilizer (*R. clarus* inoculation plus 100 kg ha^-1^ NPK 0:20:20). The fertilizer dosage (200 kg ha^-1^ NPK 0:20:20) was according with agronomic recommendations and chemicals analyses of soil in experimental areas (**Table 1**). The nitrogen (N) supply in all treatments was a commercial inoculant (Rizo Plus^®^ Rhizobacter) that contain two lines of *Bradyrhizobium japonicum* (SEMIA 5079 and SEMIA 5080) and its inoculation was according the manufacturer’s recommendation.

The treatments were arranged in a completely randomized block design with five replicates (**Supplementary Figure S2**). Each replicate consist in plots of 5 × 8 m (40 m^2^) with 10 rows with spacing 0.45 m and, a density of ten plants per linear meter (approx. 200,000 plants ha^-1^). The plots were separated by two lateral lines as edge.

#### Cotton Experiment

Cotton experiment was carried out in the harvest 2013/14 (December–June) with the following treatments: Control (Non-AMF inoculation and non-fertilizer application); Fertilizer (200 kg ha^-1^ PK 20:20 + 200 kg ha^-1^ urea); AMF (*R. clarus* inoculation plus 65 kg ha^-1^ KCl + 200 kg ha^-1^ urea); AMF + Fertilizer (*R. clarus* inoculation plus 200 kg ha^-1^ PK 20:20 + 200 kg ha^-1^ urea); and AMF + 1/2 Fertilizer (*R. clarus* inoculation plus 100 kg ha^-1^ PK 20:20 + 200 kg ha^-1^ urea). The cotton variety used was Bayer^®^ FM 975WS and the fertilizer dosage (200 kg ha^-1^ PK 20:20 + 200 kg ha^-1^ urea) was according agronomic recommendation for experimental area based in chemical analyses of soil (**Table 1**). The treatments were arranged in a completely randomized block design with five replicates as described above for soybean experiments.

### Evaluations and Harvest

The effect of *R. clarus* inoculation in soybean and cotton experiments was assessed by the quantification of effective mycorrhizal colonization of roots and their effect in nutrient uptake (N and phosphorus), biomass production (shoot dry weight), and yield grain (soybean) and lint (cotton).

In soybean experiments, roots of 10 plants per plot were sampled randomly at 30 and 80 days after emergence (DAE) to evaluate the mycorrhizal colonization. In sampled plants at 80 DAE was made the evaluations of biomass and quantification of N and P in plant tissues for variety BRS 133. The percentage of mycorrhizal colonization was estimated by the grid-line method (Giovanetti and Mosse, 1980) after fresh roots were stained (Phillips and Hayman, 1970). N and P in shoot tissues were quantified according to Murphy and Riley (1962) and Sarruge and Haag (1974), respectively. For biomass quantification, plants were cut at the ground level; the total fresh shoot height was measured and shoot dry weight was determined after drying at 50°C for 72 h. For cotton, plants and roots were sampled at 120 DAE. Five plants per treatment of each plot were randomly collected, and evaluated for AMF colonization, fresh and dry shoot height, and N and P quantification.

Relative mycorrhizal dependency (RMD) was determined by the given below (Plenchette et al., 1983).

$$(Dry\,\;weight\,\;R.\,\;clarus\,\;inoculated\,\;plants)-RMD=\frac{\left(Dry\,\;weight\,\;native\,\;mycorrhizal\,\;plants\right)}{\left(Dry\,\;weight\,\;R.\,\;clarus\,\;inoculated\,\;plants\right)}\times 100$$

Soybean grains were harvested at 120 DAE. For yield estimation were sampled four linear meters (2 m^2^) in central area of each plot; after sampling the grains were cleaned, dried, and weighted. Cotton yield was estimated at 190 DAE by counting and collecting opened bolls in 20 plants in the central rows of each plot.

### Statistical Analysis

The statistical analyses of AMF root colonization were performed using the Friedman test at a significance level of *p* ≤ 0.05. Plant growth parameters, nutrient uptake and field were analyzed by analysis of variance (ANOVA) and the Tukey test (HSD) at a significance level of *p* ≤ 0.05. The analysis was carried with BioEstat 5.0 and STATISTICA 7.0 software.

## Results

### Soybean Experiments

**FIGURE 1:**
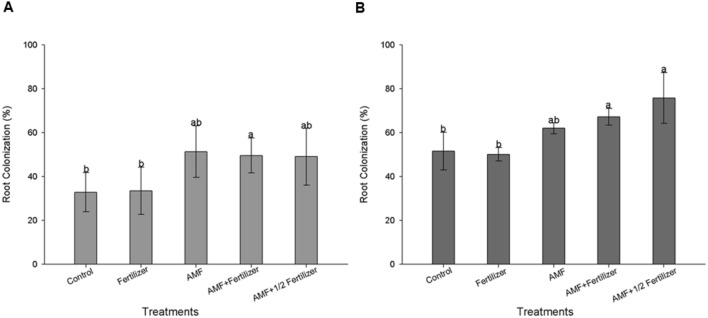
**Root colonization of soybean variety BRS 133 at 30 days **(A)** and 80 days **(B)**.** Control (Non-arbuscular mycorrhizal fungi (AMF) inoculation and non-fertilizer application); Fertilizer (200 kg ha^-1^ NPK 0:20:20); AMF (*Rhizophagus clarus* inoculation plus 65 kg ha^-1^ KCl); AMF + Fertilizer (*R. clarus* inoculation plus 200 kg ha^-1^ NPK 0:20:20); and AMF + 1/2 Fertilizer (*R. clarus* inoculation plus 100 kg ha^-1^ NPK 0:20:20). Columns followed by the same letter are not significantly different among treatments by Friedman test (*n* = 5) at *p* < 0.05. Bars represent standard error of means.

**FIGURE 2:**
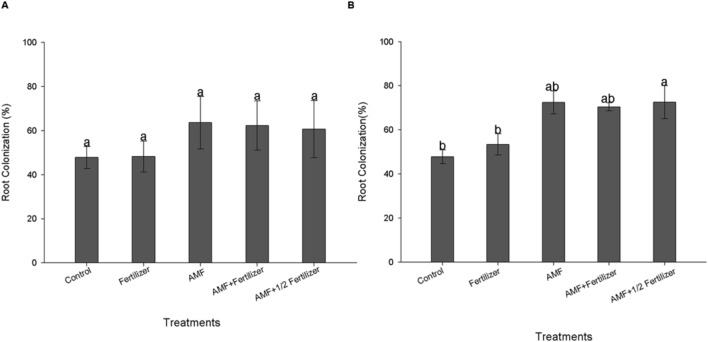
**Root colonization of soybean variety BRS 359 RR at 30 days **(A)** and 80 days **(B)**.** Control (Non-AMF inoculation and non-fertilizer application); Fertilizer (200 kg ha^-1^ NPK 0:20:20); AMF (*R. clarus* inoculation plus 65 kg ha^-1^ KCl); AMF + Fertilizer (*R. clarus* inoculation plus 200 kg ha^-1^ NPK 0:20:20); and AMF + 1/2 Fertilizer (*R. clarus* inoculation plus 100 kg ha^-1^ NPK 0:20:20). Columns followed by the same letter are not significantly different among treatments by Friedman test (*n* = 5) at *p* < 0.05. Bars represent standard error of means.

The first evaluation (30 DAE) of AMF colonization for two soybean varieties (BRS 133 and BRS 359 RR) showed that *R. clarus* inoculation increased root colonization about 20% more than non-inoculated plants; although not statistically significant, this difference indicates that inoculation have a positive effect (**Figures 1A** and **2A**). At 80 DAE the roots colonization showed higher values, around 70%, in inoculated plants with the addition of half dose of fertilizers (AMF + 1/2 Fertilizer), in this time these differences were statistically significant by Friedman test (*p* < 0,05) when compared with non-inoculated plants for two soybean varieties (**Figures 1B** and **2B**). When analyze the AMF root colonization of two soybean varieties, is possible observing that the transgenic variety BRS 359 RR had a highest early colonization (around 50% at 30 DAE) that the conventional variety BRS 133 (around 30% at 30 DAE).

The response of soybean at *R. clarus* inoculation was assessment in variety BRS 133 at 80 DAE and are show in **Table 2**. No differences were observed in plant height between the control and fertilizer or inoculated treatments. Plant biomass and nutrients (N and P) uptake showed that *R. clarus* inoculation (AMF) had the same effect that the conventional fertilization (Fertilizer) and *R. clarus* inoculation with half dose of fertilizer (AMF + 1/2 Fertilizer). These treatments presented statistic differences regarding control (Control) according Tukey test (*p* < 0.05) but not among them. In the other hand, the highest values for these variables were observed in the treatment with *R. clarus* inoculation in combination with conventional fertilization (AMF + Fertilizer). This combination increased highly N and P uptake, around 24%, when compared with soybean only fertilized.

**Table 2:** Effect of AM inoculation on height, biomass and nutrients uptake in soybean plants at 80 DAE.

Treatments	Height (cm)	Biomass (g plant^-1^)	P (mg plant ^-1^)	N (mg plant ^-1^ (×10))	RMD (%)
Control	64 ± 5^a^	21.4 ± 11^c^	52 ± 29^c^	190 ± 90^c^	–
Fertilizer	64 ± 8^a^	32.4 ± 10^ab^	82 ± 30^ab^	300 ± 80^ab^	–
AMF	62 ± 7^a^	28.9 ± 7^ab^	73 ± 13^ab^	280 ± 50^ab^	26
AMF+ Fertilizer	57 ± 6^a^	40.0 ± 8^a^	102 ± 25^a^	380 ± 90^a^	47
AMF+ ½Fertilizer	65 ± 11^a^	28.6 ± 9^ab^	75 ± 24^ab^	300 ± 90^ab^	26


*Control (Non-AMF inoculation and non-fertilizer application); Fertilizer (200 kg ha^*-1*^ NPK 0:20:20); AMF (*Rhizophagus clarus* inoculation plus 65 kg ha^*-1*^ KCl); AMF + Fertilizer (*R. clarus* inoculation plus 200 kg ha^*-1*^ NPK 0:20:20); and AMF + ½ Fertilizer (*R. clarus* inoculation plus 100 kg ha^*-1*^ NPK 0:20:20). (RMD) relative mycorrhizal dependency. Means followed by the same letter are not significantly different (*p* < 0.05) as determined by Tukey test.*

**FIGURE 3:**
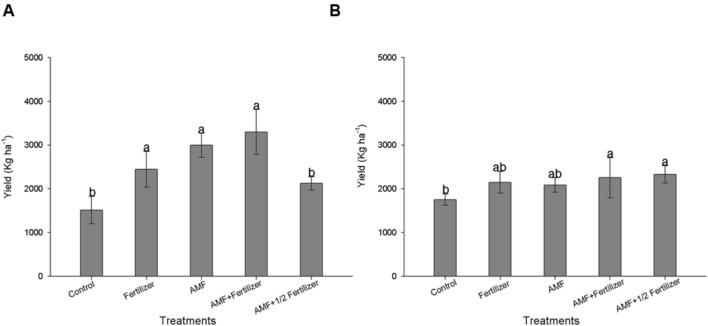
**Effect of AMF inoculation on grain yield of soybean BRS 133 **(A)** and BRS 359 RR **(B)**.** Control (Non-AMF inoculation and non-fertilizer application); Fertilizer (200 kg ha^-1^ NPK 0:20:20); AMF (*R. clarus* inoculation plus 65 kg ha^-1^ KCl); AMF + Fertilizer (*R. clarus* inoculation plus 200 kg ha^-1^ NPK 0:20:20); and AMF + 1/2 Fertilizer (*R. clarus* inoculation plus 100 kg ha^-1^ NPK 0:20:20). Columns followed by the same letter are not significantly different between treatments (*p* < 0.05) was determined by Tukey test. Bars represent standard error of means.

**FIGURE 4:**
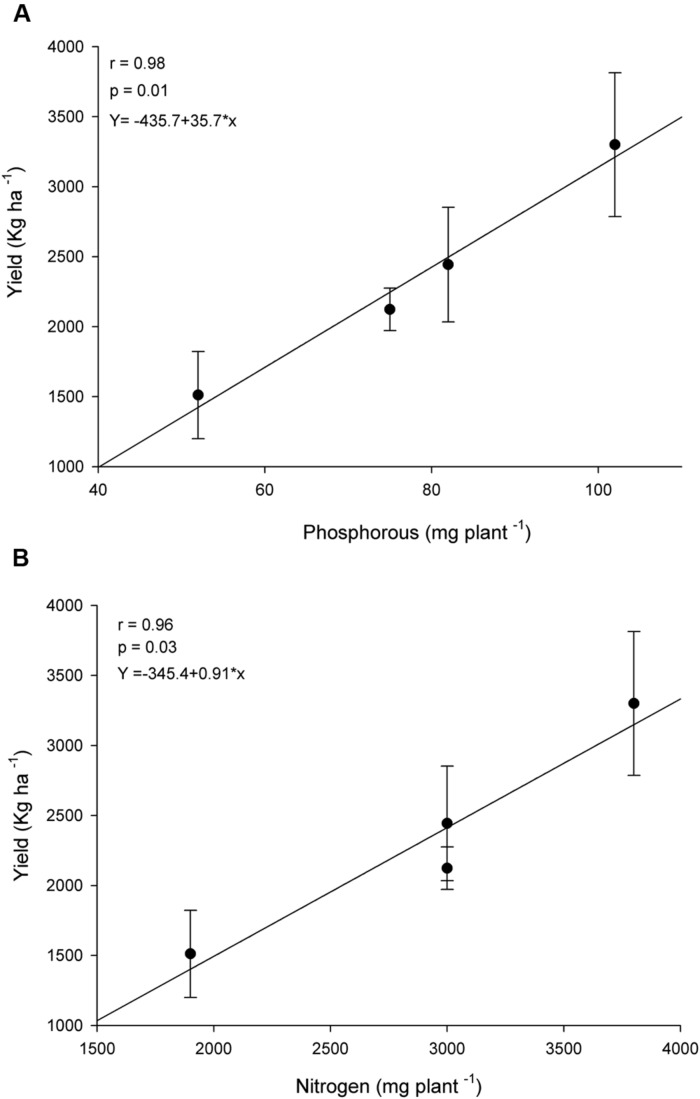
**Correlation between shoot nutrients uptake and grain yield of soybean var. BRS133.** **(A)** Phosphorous (P) uptake and grain yield. **(B)** Nitrogen (N) uptake and grain yield.

Reflecting the increase in nutrients uptake, *R. clarus* inoculum increased grain yield in cultivar BRS 133, the higher yield was observed in AMF+ Fertilizer treatment, the statistical analysis showed the yield could be equivalent between conventional fertilization, AMF and AMF + Fertilizer treatments (**Figure 3A**). Soybean BRS 359 showed the best grain yield in AMF + Fertilizer and AMF + 1/2Fertilizer treatments (**Figure 3B**). The effect of *R. clarus* inoculation showed high correlation between yield of soybean BRS 133 and P (*r* = 0.98; *p* = 0.01) and N (*r* = 0.96; *p* = 0.03) tissue contents (**Figure 4**).

### Cotton Experiment

**FIGURE 5:**
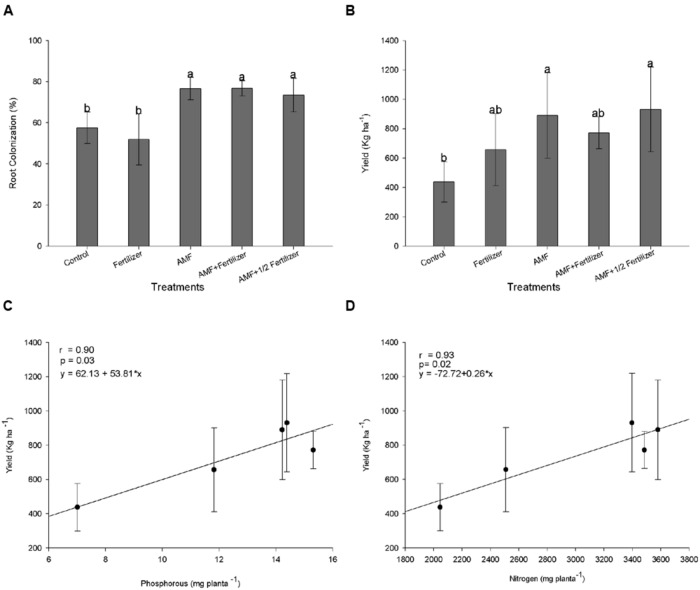
**Effect of AMF inoculation in cotton.** **(A)** Root colonization at 120 days after emergence (DAE); **(B)** lint cotton yield; **(C)** correlation between shoot P uptake and lint cotton yield; **(D)** correlation between shoot N uptake and lint cotton yield. Control (Non-AMF inoculation and non-fertilizer application); Fertilizer (200 kg ha^-1^ PK 20:20 + 200 kg ha^-1^ urea); AMF (*R. clarus* inoculation plus 65 kg ha^-1^ KCl + 200 kg ha^-1^ urea); AMF + Fertilizer (*R. clarus* inoculation plus 200 kg ha^-1^ PK 20:20 + 200 kg ha^-1^ urea); and AMF + 1/2 Fertilizer (*R. clarus* inoculation plus 100 kg ha^-1^ PK 20:20 + 200 kg ha^-1^ urea). Columns followed by the same letter are not significantly different (*p* < 0.05) between treatments by Friendman test (for root colonization) and Tukey test (for lint yield). Bars represent standard error of means for each treatment.

The *R. clarus* inoculation increased root colonization (80%) when compared with plants without inoculation (50%) at 120 DAE (**Figure 5A**) and, this difference in the colonization was statistically significant according Friedman test (*p* < 0.05), showing that just as soybean, the cotton inoculation with a *R. clarus* had a positive response.

The AMF inoculation does not show a significant effect in plant height. Others parameters as plant biomass and nutrients uptake showed differences in control plants when compared with fertilizer and AMF + Fertilizer combinations. Statistical analysis of these parameters suggest that conventional fertilization in cotton have the same effect that only AMF inoculation, in other hand the fertilization in combination with AMF inoculation (AMF + Fertilizer and AMF + 1/2/Fertilizer) not differ among them (**Table 3**). Lint cotton yield was significantly higher in plans with AMF inoculation without fertilization and in treatment with AMF inoculation with half dose of fertilizer (**Figure 5B**). Nutrients uptake showing significantly high correlation with lint cotton yield, therefore for P uptake the correlation coefficient was *r* = 0.90 (**Figure 5C**), and for N uptake *r* = 0.96 (**Figure 5D**), both statistically significant (*p* < 0.05).

**Table 3:** Effect of *R. clarus* inoculation on total shoot height, biomass, P and N shoot uptake of cotton plants at 120 DAE.

Treatments	Height (cm)	Biomass (g plant^-1^)	P (mg plant ^-1^)	N (mg plant ^-1^ (×10))	RMD (%)
Control	139.7 ± 15^a^	48 ± 12^b^	7 ± 2^b^	204.6 ± 50^b^	—
Fertilizer	137.7 ± 11^a^	59 ± 15^ab^	12 ± 4^ab^	250.8 ± 60^ab^	—
AMF	129.0 ± 14^a^	87 ± 15^a^	14 ± 2^a^	357.8 ± 50^a^	45
AMF + Fertilizer	143.0 ± 5^a^	81 ± 18^a^	15 ± 4^a^	348.4 ± 90^a^	41
AMF + 1/2 Fertilizer	136.3 ± 21^a^	82 ± 24^a^	14 ± 3^a^	339.7 ± 90^ab^	41


*Control (non-AM and non-fertilizer); Fertilizer (200 kg ha^*-1*^ PK 20:20 + 200 kg ha^*-1*^ urea); AMF (*R. clarus* plus 65 kg ha^*-1*^ PK 0:20 + 200 kg ha^*-1*^ urea); AMF + Fertilizer (*R. clarus* plus 200kg ha^*-1*^ PK 20:20 + 200 kg ha^*-1*^ urea); AMF + 1/2 Fertilizer (*R. clarus* plus 100 kg ha^*-1*^ PK 20:20 + 200 kg ha^*-1*^ urea); RMD, relative mycorrhizal dependency. Means followed by the same letter are not significantly different (*p* < 0.05) as determined by Tukey test.*

## Discussion

The inoculation of *R. clarus* increased plant growth and yield of two varieties of soybean and cotton. Apparently, the inoculum produced *in vitro* was more competitive against native AMF, since inoculated plants showed increased AMF colonization and shoot uptake of P and N. Soybean and cotton showed different responses for *R. clarus* inoculum. First, in soybean, there was a triple interaction (*Bradyrhizobium* – *R. clarus* – plant root), and the inoculum tested was infective and effective, since symbiotic bacteria were already present.

The success of AMF inoculation in agricultural soils can be determined by many factors such as species compatibility, habitat niche availability for AMF and competition with native fungi (Verbruggen et al., 2013). Compatibility is an important point for AMF inoculation, where some isolates could be host “specialists,” while others “generalists” (Öpik and Moora, 2012). The inoculum of *R. clarus* tested showed a generalist nature, since it enhanced both plant growth and yield. Accordingly, AMF that are considered plant host generalists have a high establishment rate in several crops (Öpik and Moora, 2012); the results showed that soybean and cotton were effectively colonized, indicating a low specificity by the host plants for *R. clarus*.

In the experiments, mycorrhizal colonization in control plant was around 50% indicating that the agricultural soils support an active indigenous AMF community. The adaptation of *R. clarus* and its competition capacity against indigenous AMF were high. The problem in obtaining an effective AMF inoculum to use on large scale concerns these factors exactly; the inoculum showed good infectivity and high competition capacity under field conditions.

As well known, soil P availability is one the most important factors of AMF regulation, and this characteristic is directly related to the role of P uptake in the AMF symbiosis (Smith et al., 2003; Breuillin et al., 2010; Gutjahr and Parniske, 2013). Our results showed that in soybean and cotton, the moderate soil P availability in the experimental areas (12 and 17 mg dm^-3^) did not inhibit root colonization of the native AMF population and inoculum of *R. clarus*. The effectiveness of AMF inoculation in greenhouse experiments with phosphate fertilization showed that moderate phosphate availability can allow mycorrhizal colonization, promoting plant growth (Schroeder and Janos, 2005; Taffouo et al., 2013; Xie et al., 2014), and the same responses were found in a field conditions in soybean (Maddox and Soileau, 1991; Karaca et al., 2013).

On other hand, soil P availability can be determined by soil chemical characteristics that influence phosphate solubility. In acid soils, P is less available because of immobilization, even with fertilizer applications, making it unavailable to plants (Busman et al., 2002). Rhodic Ferralsol soils in the experimental area showed low pH, where they can adsorb phosphate, and AMF has an important role in enhancing P uptake and availability, including P from chemical fertilization.

Plants with high P requirements show a high RMD index (Plenchette et al., 1983). Cotton showed a higher RMD (45%) than did soybean (26%) when inoculated with *R. clarus* in the presence or absence of fertilizer. In contrast, when P was added at the recommended dose in combination with AMF inoculation, this index decreased to 41% in cotton and increased to 47% in soybean, suggesting that the gain in biomass was related to the availability of P from the fertilizer, which *R. clarus* provided for the plant roots. Thompson et al. (2012) obtained the same results.

The finding that P and N uptake increased in both crops may be related to *R. clarus* association as observed by other authors (Allen et al., 2003; Barea et al., 2005). AMF improved plant nutrition, leading to an increase in grain yield in soybean and cotton lint production, showing a positive correlation between plant nutrition and yield. Mahanta et al. (2014) also observed a positive linear relationship between P and yield in soybean when inoculated with AMF. The effect of *R. clarus* on cotton growth and yield found here agrees with Thompson et al. (2012) who found an increase in seed cotton yield with *Glomus mosseae* inoculation. The effect of AMF inoculation. However, this is the first time that *R. clarus* inoculum obtained under axenic conditions was tested under field conditions. Ceballos et al. (2013) showed that inoculation of *Rhizophagus irregularis* increased the cassava yield in field and suggest this practice as alternative for improve this crop in several countries.

## Conclusion

The inoculum of *R. clarus* evaluated was very competitive against endogenous AMF and also increased plant growth and yield. *R. clarus* obtained *in vitro* and tested in the field was efficient in starting early AMF infection in seedlings, improving AM colonization in soybean and cotton. The inoculum of *R. clarus* helped plants to take up P from fertilizer and showed high potential for use in combination with conventional fertilization, for intensive agriculture system in large areas in tropical soils, increasing P absorption and more efficient fertilization use, this is fundamental for the actual challenge of crops production.

## Author Contributions

All authors listed, have made substantial, direct and intellectual contribution to the work, and approved it for publication.

## Conflict of Interest Statement

The authors declare that the research was conducted in the absence of any commercial or financial relationships that could be construed as a potential conflict of interest.
