# Supplementary material for: Inoculant of Arbuscular Mycorrhizal Fungi (Rhizophagus clarus) Increase Yield of Soybean and Cotton under Field Conditions
Source: Front Microbiol. 2016 May 25;7:720. doi: 10.3389/fmicb.2016.00720 (PMC4880672; doi:10.3389/fmicb.2016.00720)
Supplement: Supplementary file 1 [file Data_Sheet_1.DOCX]

Supplementary Material

Inoculant of arbuscular mycorrhizal fungi (*Rhizophagus clarus*) increase yield of soybean and cotton under field conditions

Martha Viviana Torres Cely^1^, Admilton Gonçalves de Oliveira^2^, Vanessa Fogaça de Freitas^2^, Marcelo Benite de Luca^2^, André Riedi Barazetti^2^, Igor Matheus Oliveira dos Santos^2^, Barbara Gionco^2^, Guilherme Volante Garcia^3^, Cássio Egidio Cavenaghi Prete^4^, Galdino Andrade^2^*

^1^ Laboratório de Microbiologia, Instituto de Ciências Agrarias e Ambientais, Universidade Federal de Mato Grosso, Sinop, Mato Grosso, Brazil.

^2^Laboratório de Ecologia Microbiana, Departamento de Microbiologia, Universidade Estadual de Londrina, Londrina, Paraná, Brazil.

^3^United Phosphorus Limited–UPL do Brazil, Campinas, São Paulo, Brazil.

^4^ Fazenda Escola, Departamento de Agronomia, Universidade Estadual de Londrina, Londrina, Paraná, Brazil.

*Corresponding author: Galdino Andrade, Universidade Estadual de Londrina, Centro de Ciências Biológicas, Departamento de Microbiologia. CEP 86051-990, Londrina, Brazil. andradeg@uel.br

**Supplementary Figures**


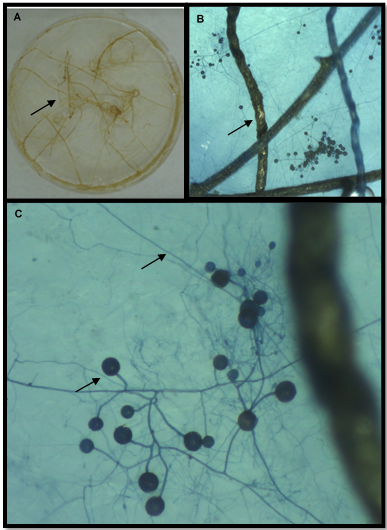


**Fig. S1** AMF *Rhizophagus clarus*. A: MRS medium plate with transformed root organ culture; B: mycorrhizal development in root organ culture; C: AMF structures hyphae and spore in vitro pure culture.


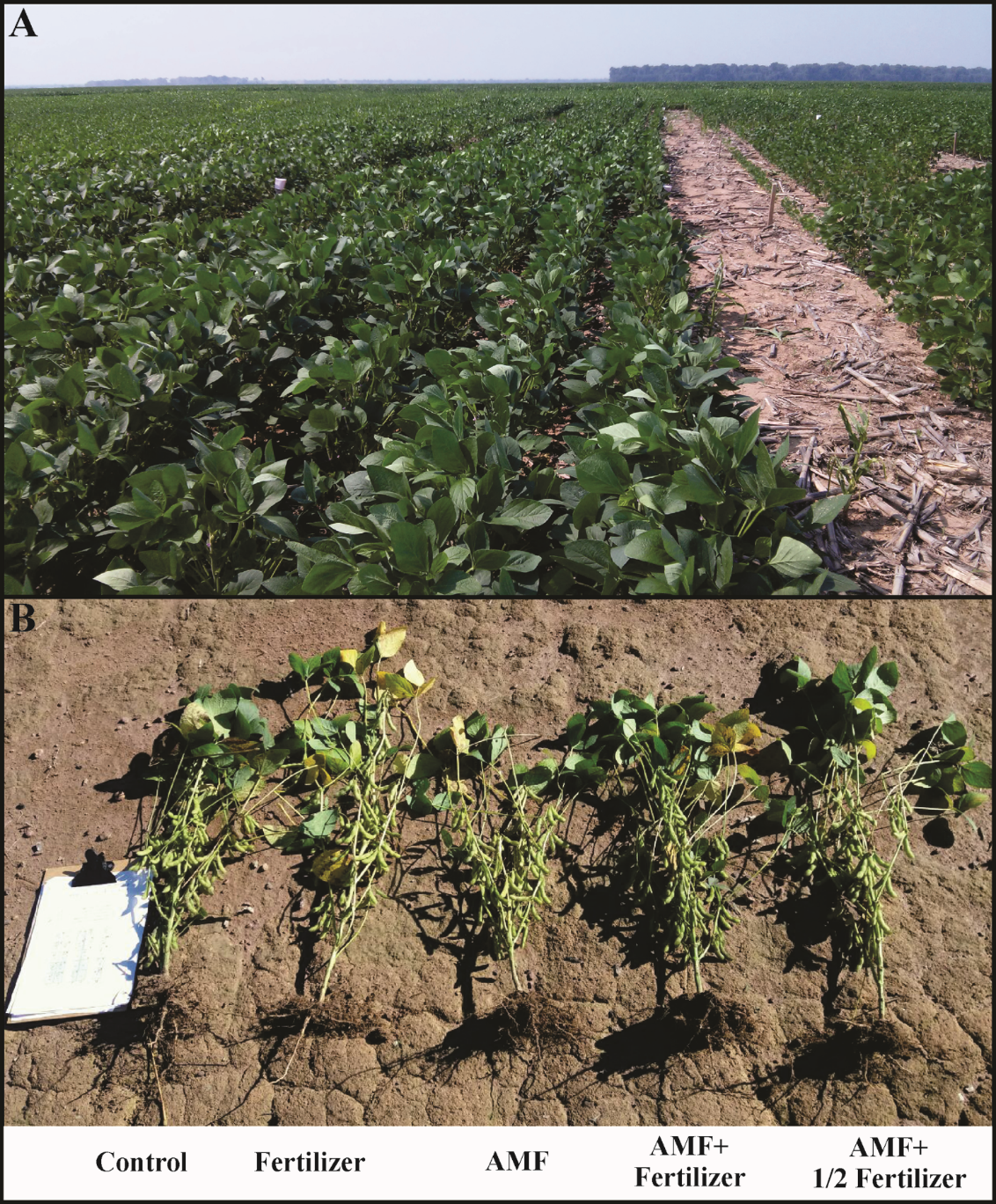


**Fig. S2** Experiments conditions in field. A: treatments arranged in blocs; B: representative plants for treatments at 60 DAE.
